# Cervical cancer screening among immigrant women in Norway- The healthcare providers’ perspectives

**DOI:** 10.1080/02813432.2018.1523986

**Published:** 2018-10-05

**Authors:** Kathy Ainul Møen, Laura Terragni, Bernadette Kumar, Esperanza Diaz

**Affiliations:** a Department of Global Public Health and Primary care, University of Bergen, Bergen, Norway;; b Norwegian Center for Minority Health Research, Norwegian Institute of Public Health, Oslo, Norway;; c Department of Nursing and Health Promotion, Faculty of Health Sciences, Oslo and Akershus University College of Applied Sciences, Oslo, Norway;; d Institute of Health and Society, Faculty of Medicine, University of Oslo, Oslo, Norway

**Keywords:** Cervical cancer screening, emigrants and immigrants, intervention, health care providers

## Abstract

**Objective:** To explore health care providers’ (HCPs) experiences regarding cervical cancer screening (CCS) among immigrant women, their strategies to facilitate these consultations and their need for further information.

**Design:** Exploratory qualitative design.

**Setting:** HCPs who perform CCS: general practitioners, midwives and private gynaecologists, working in Oslo, Norway.

**Subjects:** We interviewed 26 general practitioners, 3 midwives and 3 gynaecologists.

**Method:** Both focus groups and personal in depth semi structured interviews. Interview transcripts were analysed using a thematic analysis approach.

**Results:** Some of the HCPs’ experiences related to CCS were common for all women regardless of their immigrant background, such as the understanding of routines and responsibilities for prevention. Aspects specific for immigrant women were mainly related to organization, language, health literacy levels, culture and gender. Several strategies targeting organizational (longer consultations), language (using interpreters), health literacy (using anatomy models to explain) and culture (dealing with the expression of pain) were reported.

Most HCPs had not previously reflected upon specific challenges linked to CCS among immigrant women, thus the interviews were an eye-opener to some extent. HCPs acknowledged that they need more knowledge on immigrant women’s’ reproductive health.

**Conclusion:** HCPs’ biases, stereotypes and assumptions could be a key provider-level barrier to low uptake of CCS test among immigrants if they remained unexplored and unchallenged. HCPs need more information on reproductive health of immigrant women in addition to cultural awareness.

**Key Points**
- The participation rate of immigrant women to cervical cancer screening in Norway is low, compared to non-immigrants. This might be partly attributed to health care system and provider, and not only due to the women’s preferences. Our focus groups and interviews among health care providers show, that in addition to cultural competence and awareness, they need knowledge on reproductive health of immigrants. We recommend an intervention targeting health care providers to close the gap in cervical cancer screening.

## Introduction

In 2017, immigrant women comprised 11% of the European female population [1]. The majority of these women have migrated from Africa, Latin America and Asia, and the proportion of non-European immigrants continues to increase [2]. Many female immigrants work as caregivers or domestic helpers, and are often part of the informal labor force impacting their social position and access to resources, including access to health care [3].

Although there are more similarities than differences in the disease profiles of migrants and non-migrants, the prevalence of different types of cancer could be related to migrants’ background [4,5]. This is the case for cervical cancer, with a higher prevalence among some groups of immigrants, particularly those from East, West and Central Africa and Melanesia [6]. Although most European countries aspire to achieve equity in health care, it may not be the case for cervical cancer screening (CCS). Lower attendance to CCS programs among immigrant women might indicate inequities in access [7–10]. Our research group has also documented that this is also the case for the Norwegian CCS program [11].

In Norway, every legally residing individual is entitled to a general practitioner (GP). Since1995, all women between 25 and 69 years receive a letter from the Norwegian cancer registry every three years inviting them to make an appointment with their GP for a CCS test. It is the GP who usually performs the CCS. As GPs are private practitioners, a co-payment from patients is usually required. Midwives provide free services for pregnant women and children up to pre-school age at health clinics, and their appointments with patients are usually longer than the typical GP appointment. Recently, a few midwives have begun to perform CCS tests.

Gynaecological consultations raise several challenges for both patients and providers and could be even more pronounced when the patient is an immigrant woman. Previous studies have focused on barriers for users [8,12,13]. Our recent study concurs [14] revealing barriers for attendance to CCS among immigrant women. Our findings relate to individual attitudes and perceptions on CCS; such as poor knowledge about the disease, lack of perceived necessity, language barriers or fear of pain/procedural discomfort and receiving bad news related to the test. Our study also pointed out sociocultural barriers such as stigma attached to the disease, female circumcision, or the shame for unmarried women undertaking a gynaecological examination. Our findings concur with those from Canada regarding barriers such as poor knowledge about cancer and its risk factors and lack of open discussion about issues related to female reproductive organs [15]. Another Norwegian study [16] also revealed barriers related to navigating health care system in a new country, although this was not specific for CCS.

According to the literature, health care providers (HCPs) could improve the attendance to CCS among immigrants by helping women to understand the importance of regular screening and the benefits of the CCS test [17,18]. However, immigrant women from Somalia and Pakistan report [14] that they neither receive the invitation letters from Norwegian cancer registry, nor were asked by their GPs about CCS in our recent study. Few studies have described barriers at the physicians’ and system level [17,19] and studies on HCPs’ perspectives and roles are scarce [20–22].

The aim of this study was firstly, to understand the HCPs’ experiences related to gynaecological examinations and CCS among immigrant women, secondly to learn what kind of strategies HCPs already used to overcome any barriers encountered in these consultations, and finally their need for additional information or assessment tools.

## Method

### Design

This study took place in Oslo, Norway, and has an exploratory qualitative research design [23]. Data were gathered through focus groups and personal semi-structured interviews.

### Participant selection and recruitment

As mentioned earlier, performing CCS tests is one of the GPs’ tasks. However, GPs refer women to gynaecologists in case of complexity. It is not the practice for midwives to undertake CCS, but recently as part of an experimental project, a few of them have begun to do so. Therefore, we have included some gynaecologists and midwives also as participants.

GPs attend two kinds of educational meeting groups: i) compulsory groups in order to become specialists for a two-year period, and ii) thematic courses to obtain or renew their specialty. Two supervisors of these compulsory groups were contacted by e-mail using the authors network (KAM, ED). The GPs participating were relatively young, most worked in Oslo and not known to us previously. Furthermore, we contacted the supervisor of one thematic course, comprising participants from different age groups and working in different places in Norway. All supervisors and GPs in the three groups agreed to participate in the study.

Gynaecologists and midwives were invited to the project by leaders of the midwives’ association and gynaecologists’ association. Although we intended to conduct focus groups for all the professions, the numbers of those willing to participate were few among private gynaecologists and midwives. Therefore, we conducted three focus groups (FG) among GPs and two personal semi-structured interviews with gynaecologists (one interview was with 2 participants) and two personal semi-structured interviews with midwives (one interview was with 2 participants). The first and the last author interviewed a total of 33 participants, 27 GPs, 3 gynaecologists and 3 midwives from November 2015 to March 2016 in different areas in Oslo.

### Data collection and analysis

The interview guide covered three main topics: 1. HCPs’ experiences regarding gynaecological examinations and CCS, 2. their strategies (if any) to make these consultations work well and 3. their need for more information or other materials in order to improve uptake to CCS among immigrant women.

The interviews were conducted in Norwegian, recorded and transcribed verbatim and anonymized. Interviews were analyzed using thematic analysis [24]. Themes were developed using a hybrid approach combining deductive and inductive coding [25]. Codes for the analysis were developed after an initial reading of all the transcripts and were based on the main interview questions, prior research, and emergent concepts from the current data. To develop the codes, three of the authors (KAM, LT and ED) independently reviewed two focus group transcripts. These initial codes were discussed among the authors and a codebook was developed. The codes were further refined during coding of subsequent transcripts. Codes were successively aggregated in overreaching themes. Quotes were selected to illustrate the results.

### Ethical aspects

Written informed consent was obtained from every participant before the focus group or interview started. The project (2015/1156) was approved by the Norwegian Regional Committees for Medical and Health Research Ethics.

## Results

The characteristics of the 32 participants are summarized in Table 1. The length of their professional experience varied from a few months to thirty years. Most GPs and all the recruited gynaecologists and midwives had extensive experience with immigrants.

**Table 1.** Characteristic of participants.

	GPs [27]	Midwives [3]	Gynecologists [3]
Age			
30–40	18	0	0
41–50	2	2	0
51–60	6	1	2
61–70	1	0	1
Sex			
Female	17	3	2
Male	10	0	1
Immigrant background			
Norwegian	20	3	3
Non-Norwegian	7	0	0
Length of practice			
<10 years	17	0	0
>10 years	10	3	3

## Health care providers’ experience regarding gynaecological examinations and cervical cancer screening

Most of the participants had contact with immigrant patients on a regular basis, however, very few had reflected previously upon specific challenges linked to CCS among this group. A typical comment at the beginning or the end of the interviews was:

I have never thought about this before – that immigrant women do not come for cervical cancer screening test or that they might have different prevalence/risk for cervical cancer GP2(F2FG1).

For many, these interviews were to some extent an eye-opener. Through the analyses of the data, HCPs’ experiences were classified into two broad groups: i) HCPs’ perspectives that are related to all women and ii) Perspectives that are specific for immigrant women.

### Perspectives related to all women

#### Routines and ‘not my responsibility’

GPs explained that they usually did not invite women (Norwegian or immigrant) to the CCS test on a regularly basis. Very few GPs, especially females, raised the subject with every woman, regardless of immigrant background or type of consultation. Some raised the subject during consultations related to contraception, pregnancy or routine post-natal check-ups.

The attitude of some GPs was that the CCS test is not compulsory, it is the women’s responsibility to make an appointment with their GPs and ensure that its done. As one participant shared his view:

I never ask unless it's about bleeding or something like that. They get invitation-letters from Cancer Registry every three years and reminder-letters. I think that this is something they should take responsibility for GP4(F4FG1).

### Perspectives related specifically to immigrant women

In addition to the above mentioned common perspectives, other themes emerged during the FG that were specific for immigrant women. We have grouped these into (i) organizational, (ii) language and health literacy, and (iii) culture and gender.

#### Organizational

Most HCPs had experienced that immigrant women neither made specific appointments for CCS, nor raised the issue themselves upon receipt of the Cancer Registry’s invitation letter. As several issues were raised often in one consultation, CCS test was either not prioritized or forgotten. Thus, GPs experienced the usual time constraints as a bigger obstacle for their meetings with immigrant women. As one GP said:

(….) there are many immigrant women who have several somatic illnesses and their list of issues is long when they come to us. The fact is that consultation is over before we come to CCS, it will be either postponed from time to time or forgotten before you reach the bottom of the list GP10(F7FG1).

However, some GPs gave a more nuanced picture of their experiences regarding consultations with immigrants. Women from Eastern Europe were used to taking the CCS test with their gynaecologist in their home countries. These women often asked their Norwegian GPs for direct referrals to a gynaecologist.

It is true that many immigrant women are used to go to gynaecologists and they may not realize that these are tests that we GPs do here in Norway (…) GP6(F5FG1).

Some GPs reflected upon the possibility of taking the CCS test within primary care, but out of the GP office referring to some midwives in different parts of Oslo who have recently started to perform CCS test. Accordingly, the interviewed midwives confirmed that in their experience immigrant women have low threshold to come to them for CCS test.

From my experience, I have the impression that because GPs do not have the same function as us and GPs may not have enough time, the women really have confidence in us and want to come to us because we have time, this is a 100% female work-place (laughs) and the CCS test is free of charge JM2(F19PI2).

#### Language and health literacy

HCPs described that language is important for better communication. As one HCP told us;

Language is really a key, (…) I often have the impression that also immigrant women could actually be open about sex and intimate things (…) GN2(F22PI4).

HCPs explained that most of immigrant women, especially first generation, had low health literacy. This resulted in time-consuming consultations.

It is in a way very difficult to know where one can start when you have 20 minutes available. We can hardly let it become an anatomy lecture every time GN2(F22PI4).

#### Cultural aspects and gender

As explained above, GPs tended not to ask any women about CCS test, and a few GPs thought that it was generally not their responsibility to ask women about CCS test since they got the invitation letter from the Cancer Registry. However, some GPs seemed to have an even higher threshold to ask when the patient was an immigrant woman belonging to another culture. As one of the female GPs mentioned:

I think the threshold to take the initiative to ask about CCS is higher the more different the woman is from me. For example, clothes, just think that you're going to get rid of that ‘burka’, it is a signal about the type of shyness/embarrassment one must pass through GP19(F12FG2).

Some participants also had experienced that some groups of immigrant women often expressed more pain under gynaecological examinations, and this was understood as cultural. As one participant said:

I have a slight impression that women from particular countries, for example, African and some Asian countries, seem like they express more pain and anxiety about such type of examinations. I was wondering if this was something to do with the culture (…) GP21(F14FG3).

Differences regarding attitudes and behaviour of immigrants and their offsprings were also observed.

Potential barriers in the interaction between women and male HCPs were often brought up during the interviews. According to male GPs’ experience, immigrant women expressed their preference to take the CCS test with a female physician more often than Norwegian women. Male GPs reported therefore that the threshold to refer the women to a female colleague or female gynaecologist was low:

No, I do not take many CCS tests, eh, it's really because they probably want a female doctor who does this or to go to a gynaecologist GP12(M5FG2).

Male HCPs also indicated that they experienced discomfort in taking up the topic of CCS test with immigrant women.

It's a cultural barrier, especially for me who is a male, (…) when it comes to how to relate to women who have a different woman-man relationship than what we have in the West GN3(M11PI4).

However, the same male gynaecologist also explained that once he got to know the immigrant women and established a good relationship, some of them continued to make appointments with him despite gender. This experience was similar to a male GP.

I think I should establish some kind of trust with the patient, ‘is it okay, are you prepared to take the CCS test today, it may be that you are not ready for it today, but I will set up a new appointment’ (…), but I think trust is important before I do a gynaecological examination.

## Health care providers’ strategies to overcome challenges

### Organizational

Investing enough time was key for facilitating the consultations. Some spent more time when they took CCS test or gynaecological examinations. This helped also when they explained medical findings to the women. One female GP told us the following:

I experience that often it's hard to perform a gynaecological exam, I use more time to talk and explain in these situations GP21(F14FG3).

Gynaecological examination and CCS are sensitive issues, that are further complicated for both immigrant women and their GPs when there is a male interpreter in the same room or family members as interpreters. There were different strategies to overcome these challenges, as one of the GPs explained:

What I did was that I explained him (male interpreter) inside my office first what we were going to do, and I asked him to wait outside when I took the CCS test, I fetched him when we were done (…) GP22(F14FG3).

### Language and health literacy

HCPs tried to use interpreters, speak slower, use simple words and sentences, and sometimes used body-language. They often used anatomy models and drawings to communicate. As one midwife told us:

We have anatomy models; a doll, pelvis or spinal column, so I sit so many times with that doll or the pelvis and explain what happens when they give birth JM3(F20FG5).

Most HCPs agreed that they often simplified or even skipped explanations due to language difficulties or assumed a lack of basic knowledge among women about their own body as compared to non-immigrants. In addition, one gynaecologist mentioned that she took CCS test sometimes without informing the women due to lack of time.

### Culture

Dialogue about cultural issues between HCPs and the patient regarding gynaecological consultations were seldom described in the focus groups. Some HCPs, often those with contact with many immigrants, had tried to adapt to what they understood as cultural or religious barriers. A midwife shared her experience as follows:

Norwegian women may be satisfied with hormonal IUD, but we found out that it may not be suitable for many Muslim women. So, we read what was said in Quran about contraception and we mapped out carefully before we started guiding them which method was acceptable in a way. Culture and religion are very important, so what matters to them is important for us (…) JM1(F18FG4).

## Health professional’s preferences on how to get more information

After sharing their experiences and strategies, all participants identified the need for more information about this subject both for themselves and for other colleagues. We discussed the possibilities to provide this information in the future, such as courses, visits to GP offices or written information such as e-mails, brochures, letters and posters. Given a choice, most of them preferred short visits by experts in this field during lunch or morning meetings at the GP offices. In addition, giving information to the women directly through other channels was mentioned by all.

## Discussion

Despite the lack of attention given by HCPs to possible challenges in gynaecological consultations and CCS among immigrant women, several experiences were shared through focus group reflections by all three professions. The inclusion of gynaecologists and midwives in addition to GPs enriched our perspectives, mainly regarding organizational and gender-barriers. Some of the experiences shared were applicable for all women, while others were specific for immigrant women. While HCPs shared with us strategies to facilitate consultations with immigrant women, they also reflected upon their need for more information on migrant health to improve their case management.

Previous studies have explored HCPs’ perspectives on immigrant women’s health [26–29], but very few have explored the specific challenges of gynaecological consultations and CCS [20,21]. Consistent with earlier findings [17,19,21], the HCPs considered time-constraints, communication and cultural discordance as challenges to varying degrees. Use of interpreters for gynaecological examinations, in particular a man, came up as a sensitive issue, and could also be linked to other challenges related to confidentiality and vulnerability. Additionally, low health literacy levels often co-exist with language challenges and was also mentioned by several informants. However, our study adds some new knowledge by suggesting that organizational challenges might be as important as cultural differences in the HCP’s performance.

The main challenge for HCPs was that CCS was seldom on the agenda for the consultation. On the one hand, immigrant women to a lesser degree than non-immigrants took the responsibility for making CCS appointment themselves. On the other hand, the HCPs seldom informed the women about CCS either, as some previous studies have described [17,19,30]. Although the lack of CCS on the agenda was not specific for immigrants, other factors seemed to make the informational task more difficult for the GPs when working with immigrants as compared to non-immigrants.

Organization of time seemed to be a key issue. Due to additional time constraints for the consultations with immigrants because of different language, health literacy levels and expectations for the consultation, GPs claimed that taking the CCS test was more often forgotten for immigrants. Time constraint in GP consultations was thus considered by GPs as a more important barrier in consultations with immigrants as compared to the majority population. Although GPs undertake most of the CCS tests, there is an on-going discussion regarding the role of midwives in Norway for this task, given that they have longer consultation time and are already in contact with women in relation to pre- and postnatal care.

Midwives included in this study had already started to take CCS tests, mostly as pilot projects. They had longer consultations and seemed to engage in more partnership-building with the immigrant women. In our study, GPs raised the issue that the CCS test should be conducted elsewhere within the health system, in particular with midwives whose consultations are free of charge and with more time to talk and build a better interpersonal relationship with the women. While this concurs with a study from Finland [31], the midwives recruited to our study worked with a greater proportion of immigrant women and might not be representative for midwives working with the general population. Therefore, the results should be interpreted with caution. However, our findings clearly point to organizational matters as key to improve uptake to CCS programs, and the benefits and possible pitfalls of midwives taking CCS test should be further evaluated.

Although the participants shared with us several challenges they encountered and how they tried to manage in the best possible way, a general discomfort regarding religion and cultural themes related to gynaecological consultations came up in all the groups and has been previously described [21]. Culture is a complex social phenomenon that can include knowledge, experience, belief, values, actions, attitudes, meanings, religion, notions of time, spatial relations and concepts of the universe for a group of people [32]. Furthermore, culture is not static and there are different degrees of acculturation within immigrant groups. In addition, HCPs, regardless of gender, should be aware of his or her own cultural beliefs, perceptions and values [33].

In the intercultural communication process, when people of dissimilar cultural backgrounds interact with one another, they are likely to rely on their preconceived stereotypes concerning certain cultural groups [34]. In our study, GPs seemed to be too busy to raise and reflect upon their own cultural and socioeconomic background, and eventually their stereotypes, bias or prejudices towards patients with different backgrounds. As such, many challenges were experienced as only related to the patient’s cultural background, and the HCPs seemed to have several non- empirically tested assumptions of what women expected, especially regarding gynaecological issues. In this regard, a novel finding of our study is that HCPs’ biases, stereotypes and assumptions could be a key provider-level barrier to low uptake of CCS test among immigrants if they remained unexplored and unchallenged. In agreement to this, previous studies show that immigrant women prefer physicians who speak their language and from their own immigrant groups for reproductive consultations [15].

Furthermore, the interaction between HCPs in European countries and immigrants might in itself be a barrier to utilization of the health care system [35], not only based on cultural differences but also on other sociocultural differences. As previous studies have shown [27], male providers could be an obstacle for some women seeking help, but according to two of the male participants (one gynaecologist and one GP), the gender difference between male HCP and his female immigrant patients could be bridged by building a good physician-patient relationship over a period of time and being aware of the cultural background of the patient. Acculturation and time trends regarding gender were also mentioned by some HCPs when they referred to an increasing number of women attending the consultations for gynaecological examinations without their husbands.

The main strength of this article is the specific focus on gynaecological consultations and CCS for women with immigrant background from the perspective of all involved HCPs. The inclusion of GPs, gynaecologists and midwives give us insight on different perspectives of HCP and possible future implementations that could make CCS more efficient. As GP participants were selected from continued education groups and not individually, we avoided those particularly interested in either immigrant or reproductive health. Through the three focus groups among GPs we reached information saturation. Additionally, the four personal interviews gave us in-depth information that can sometimes be difficult to achieve in groups when it comes to sensitive issues.

However, both the gynaecologists and midwives participating in the study were more likely to be self-selected because of the study theme, as compared to GPs. Almost all HCPs were from urban areas, which might be a limitation since living in rural areas has previously been related to higher attendance to CCS [11]. As a common limitation in this type of study, HCPs shared their perceptions about immigrant women and CCS, but validating actual practice and implementation of strategies was beyond the scope of our study.

## Conclusion

The gap in uptake for CCS test between immigrants and non-immigrants seems not only to be caused by the immigrant women’s preferences, but also by provider level barriers that are organizational, including factors such as HCPs’ biases, stereotypes and assumptions and lack of knowledge. In addition to cultural competence, there is a need for HCPs for knowledge on immigrant reproductive health. In the light of our findings, we believe that educating HCPs and students about cultural sensitivity and awareness is important in order to respond to increasing diversity. Besides practicing patient-centred communication, the HCP, regardless of gender, should be aware of his or her own cultural beliefs, perceptions and values. We recommend an intervention targeting HCPs to close this gap in the attendance of CCS.
